# Enhancing quality water, good sanitation, and proper hygiene is the panacea to diarrhea control and the attainment of some related sustainable development goals: A review

**DOI:** 10.1097/MD.0000000000039578

**Published:** 2024-09-20

**Authors:** Esther Ugo Alum, Emmanuel Ifeanyi Obeagu, Okechukwu Paul-Chima Ugwu

**Affiliations:** a Department of Research Publication, Kampala International University, Kampala, Uganda; b Department of Medical Laboratory Science, Kampala International University, Kampala, Uganda.

**Keywords:** diarrhea, hand hygiene, Nations Sustainable Development Goal, sanitation, WASH, water quality

## Abstract

Diarrhea is the second major source of ill health and pediatric death globally. It accounts for over 90% of loss of life in infants especially those below 5 years old in developing nations. Lack of quality water and good sanitation is the principal root of diarrhea. Poor nutritional status also increases the incidence of diarrhea. The United Nations Sustainable Development Goal (SDG) number 3 targets to put a stop to avoidable deaths among newborns and infants under 5 years old by the year 2030. Interestingly, SDG number 6 targets to ensure all-round and fair access to safe quality portable water, good sanitation, and proper hygiene for everyone by the year 2030. Unfortunately, South Asia and sub-Saharan African regions are centers of limited improved water and good sanitation facilities, thus explaining the increased morbidity and loss of life orchestrated by diarrhea in young children in these areas. Therefore, enhancing water quality, good sanitation, and proper hygiene is a pivotal interposition strategy to improve children’s health and well-being and achieve SDG 3, especially in the fight against diarrhea. Due to the interrelated relationship between the SDGs, improving water quality, sanitation, and hygiene (SDG 6) appears to be the foundation for achieving other goals such as reducing malnutrition (SDG 2), eradicating poverty in children (SDG 1), building good working conditions (SDG 8), protecting the environment and climatic variations (SDG 13).

## 1. Introduction

Diarrhea is the passing of free or watery stools 3 or more times within 24 hours (or more frequent passage that is one-of-a-kind from ordinary) accompanied by abdominal symptoms such as cramping, bulking, gas, and dehydration.^[1]^ Worldwide, 1.7 billion circumstances of diarrhea happen every 12 months, resulting in over 525,000 pediatric deaths each year. About 8% of all deaths in children under 5 years of age are attributed to diarrhea.^[1,2]^ Diarrhea is the second foremost source of illness and death in children globally and is liable for over 90% of loss of life in children under 5 years of age in developing nations.^[2]^ In developing countries, children below 3 years old have an average of 3 incidences of diarrhea every 12 months. Sub-Saharan Africa (SSA) has the highest child mortality rate from diarrhea. Africa and South Asia are the regions where more than 80% of childhood deaths orchestrated by diarrhea occur.^[1]^ From death records, the 5 countries with the most common diarrheal deaths are India, Nigeria, the Democratic Republic of Congo, Ethiopia, and Pakistan.^[3]^ Diarrhea is easily treatable and preventable.^[4]^ Diarrhea can harm a child’s cognitive development and progress.^[5]^ As a result, paying precise attention to infants in this age bracket will have a clear impact on the child mortality rate. Poor quality domestic water and poor sanitation practices are the main precursors of diarrhea, accounting for 72% and 56% of loss of life caused by diarrhea in young children below 5 years old, respectively.^[3]^ Poor nutritional status also increases the incidence of diarrhea.^[6]^ The incidence of diarrhea differs significantly subject to the age of the child. Younger children are mostly affected as a result of their immature immune structures.^[7]^ Among the measures to actualize the accomplishment of the United Nations (UN) Sustainable Development Goal (SDG) 3 by 2030, focused especially on forestalling deaths in newborns and kids below 5 years old,^[8]^ efforts at minimizing diarrhea should be intensified considering the fact that diarrhea is one of the main cause of loss of life in infants. SDG 6 targets to attain all-inclusive access to quality portable water, good sanitation, and proper hygiene for everyone by 2030.^[9]^ However, in 2020, approximately 26% and 46% of the global population are deficient in enhanced drinking water and good sanitation, respectively. It is estimated that by 2030 billions of humankind will be deficient in enhanced water and proper sanitation unless progress is quadruplicated.^[10]^ Furthermore, there are remarkable imbalances in the availability of clean domestic water and good sanitation facilities between localities and between urban and rural settings. Individuals who reside in urban areas have greater access to enhanced sanitation and clean water than their counterparts in rural settings.^[11]^ In fact, rural settings in South Asia and SSA are the hub of individuals who lack access to enhanced domestic water and good sanitation services.^[12,13]^ In a nutshell, only 2 out of 10 individuals in rural settings have good-quality water.^[14]^ An unfair incidence of diarrheal morbidity and mortality occurs in children below 5 years old in developing regions, wherein adequate health care, water, and improvements in cleanliness and hygiene are limited.^[15]^ Thus, enhancing the availability of quality water, and proper cleanliness, are vital intervention strategies toward enriching children’s health care and actualization of SDG 3 and other interconnected SDGs. The goal of this review is to build a strong and convincing case for water quality improvement, sanitation infrastructure, and hygiene practices in the prevention and control of diarrhea. In addition, it looks at the ways in which the interventions help in pursuit of the goals of the SDGs related to health, water, and sanitation, in particular. The ultimate goal of the review is to evaluate and synthesize existing research findings and provide clear-cut answers to questions on effective strategies, obstacles, and solutions for the realization of these objectives and sustainable development implementation on a global scale.

## 2. Methodology

### 2.1. Research design

The methodology employed in this narrative review comprised a thorough literature search and synthesis with the aim to determine the association between the implementation on a large scale of water quality, sanitation and hygiene interventions and the reduction of diarrhea incidence in children under 5 years old in developing countries. Systematic approach was basically used to select only those researches that were in line with the predefined criteria. The analysis aimed at yielding not only the effectiveness of water quality, sanitation, and hygiene (WASH) interventions into diarrhea morbidity and mortality, but also their implications to SDG 3 and other SDGs which are all connected.

### 2.2. Data collection methods

A comprehensive electronic database search including PubMed, Google Scholar, and Web of Science was done using set keywords: “water quality,” “water sanitation,” “hygiene,” “child health,” diarrhea and all WASH related interventions. Also, manual searching was done by reviewing reference lists of relevant articles and reports. The search was conducted in English language, only the studies carried out from January 2012 to December 2023 were examined. Studies examining how WASH interventions reduced the level of diarrhea in young children <5 years old in low- and middle-income countries were reviewed.

### 2.3. Inclusion criteria

Studies conducted in low- and middle-income countries.

Studies assessing the impact of WASH interventions on diarrhea incidence in children under 5 years old.

Studies published in peer-reviewed journals or reputable reports.

Studies published in English language from January 2000 to December 2023.

### 2.4. Exclusion criteria

Studies conducted in high-income countries.

Studies not focusing on diarrhea morbidity or mortality.

Studies not reporting relevant outcomes or measures.

Studies published before January 2012 or after December 2023.

### 2.5. Strengths and limitations

This narrative review has various strengths including the thorough search strategy, strict selection criteria, and coherent aggregation of data. Nevertheless, these limitations are to be expected as the methodology is prone to such biases as publication bias, different types of study designs and methodologies, and the populations and environments operated by various studies. However, a weakness of the study was that only high-quality reports were studied and the findings that were presented to the public transparently.

### 2.6. Ethical considerations

The ethical approval was not mandated for this narrative review because it was the research based on the study of the existent literature and did not include the contact with the individuals. On the other hand, ethical principles that include respect of intellectual property (copyright laws) as well as citation of sources were maintained all over the review process.

### 2.7. Analysis techniques

The studies included were evaluated critically in terms of their quality and the way they relate to the research question. Extraction of data was performed, that is key information include study design, sample size, population parameters, intervention details and outcomes related to diarrhea incidence is collected. A narrative synthesis was next used to integrate the submissions of the selected studies and to determine the recurrent themes and patterns across the literature. The process comprised the development of structure and groups, logic of differences, and interpretation of the whole data set.

### 2.8. Research question

**Figure 1.**
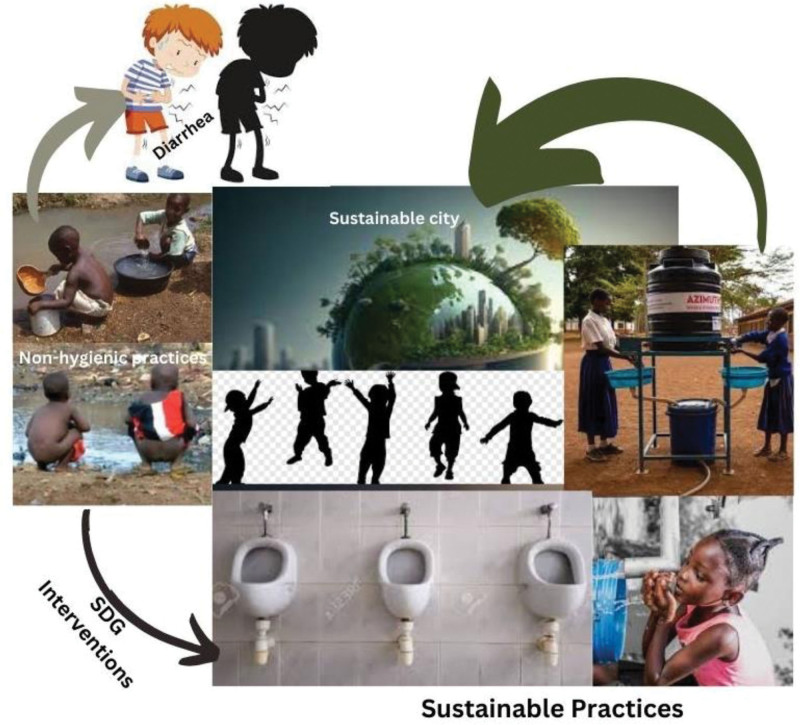
SDG interventions and sustainable practices.

**Figure 2.**
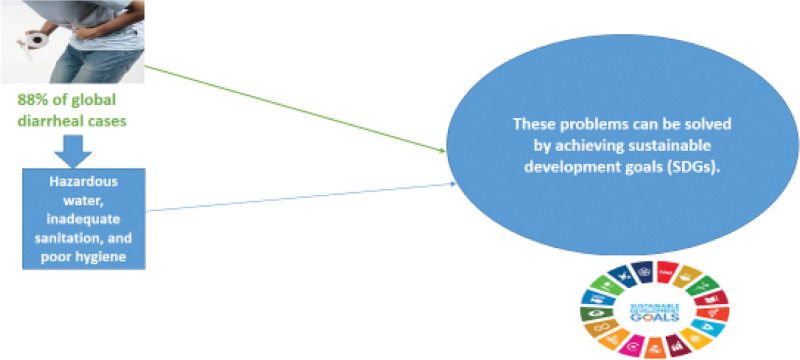
SDG 3 target of ensuring health and well-being of all.

**Figure 3.**
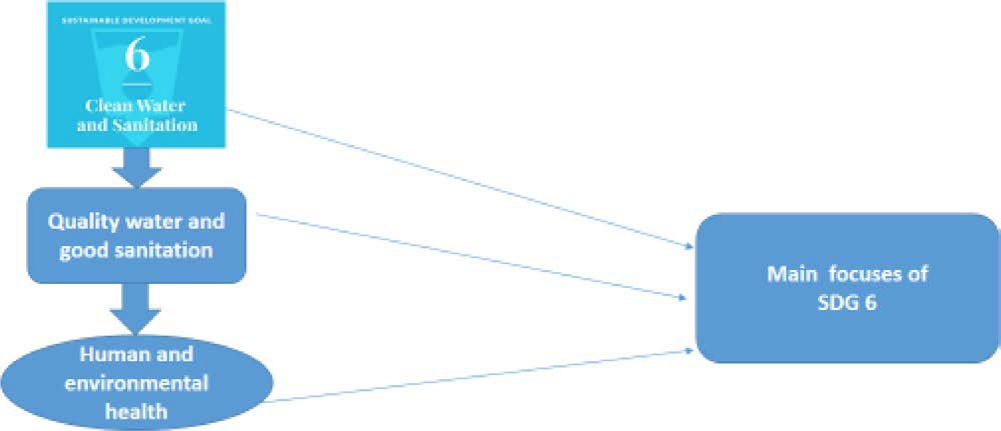
Importance of water and sanitation in achieving SDG 6.

**Figure 4.**
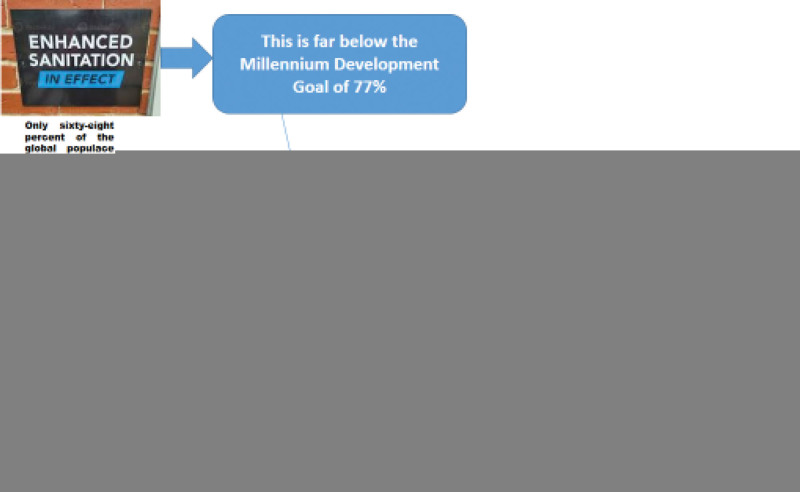
Enhancing access to good sanitation.

This narrative review aims to explore the effectiveness of WASH interventions in reducing the incidence of diarrhea in children under 5 years old in low- and middle-income countries. Specifically, the research question guiding this study is: “What is the impact of WASH interventions on diarrhea morbidity and mortality in children under 5 years old in developing nations, and how does this contribute to achieving Sustainable Development Goal 3 (SDG 3) and other interconnected SDGs?” Figure 1 shows SDG interventions and sustainable practices, Figure 2 shows SDG 3 target of ensuring health and well-being of all, Figure 3 shows Importance of water and sanitation in achieving SDG 6 and Figure 4 shows Enhancing access to good sanitation (Provided by the authors).

## 3. Enhancing quality WASH

About 88% of global diarrheal cases are attributed to hazardous water, inadequate sanitation, and poor hygiene.^[16]^ A brighter, more sustainable future for everyone can be attained through achieving SDGs. The year 2015 saw a gathering of international leaders (UN) to discuss issues such as peace, gender inequality, poverty, climate change, and health issues. The SDGs, a set of 17 intertwined goals aimed at creating a better world by 2030, were born out of this gathering. To attain the SDG 3 target of ensuring health and well-being for all, a robust dedication has been made to get rid of AIDS, tuberculosis, malaria, and other infectious illnesses by the year 2030. Furthermore, it aims to provide all-round healthcare where everybody has access to medicines and vaccines that are both safe and potent. With all nations intending to reduce newborn deaths by a minimum of 12 in 1000 live births and under-5 deaths by at least 25 in 1000 live births by the year 2030, SDG 3 forestalls needless deaths of neonates and infants under 5 years of age. A critical element of this process is improving access to affordable medicines in addition to boosting funds allocated for vaccine research.^[17]^

### 3.1. The importance of water and sanitation in achieving other SDGs

Quality water and good sanitation for everyone are the main focus of SDG 6. Quality water and good sanitation are crucial for both human and environmental health. SDG 6 focuses on the provision of quality and sustainable portable water, good sanitation, and proper hygiene.^[9]^ For improvements in other SDG target areas like good nutrition, quality education, good health, and gender fairness to be achieved, enhancements in quality drinking water, good sanitation, and proper hygiene are important. Each year, diseases connected to poor sanitation, hygiene, and low-quality drinking water cause millions of untimely deaths.^[18]^ Young children are the most afflicted and WASH-related diseases remain one of the principal reasons for loss of life in infants under 5 years old and facilitate malnutrition and developmental delays. Every year, 300,000 children under 5 years old lose their lives from diarrhea due to poor quality water, good sanitation, and hygiene. Notwithstanding tremendous advances, 2.2 billion people worldwide lack quality drinking water sources. Over 50% of the global population lacks good sanitation facilities.^[1]^ One-third of residents of low-income countries have difficulty accessing clean water. Despite the tremendous reduction in the number of people across the globe who lack access to improved water sources over the past 25 years, the deprived nations are still faced with the plight of inadequate water and poor sanitation facilities. About a quarter of residents in low-income nations had access to enhanced sanitation while over half of residents in middle-income countries had access to improved sanitation in 2015.^[16]^ The availability of quality water supply and sanitation are affiliated with climate change, and water resources management. The availability of clean water and adequate sanitation are crucial for advancement in other sectors like agriculture, sustainable energy, disaster flexibility, human health, the environment, and economic progress. In many developing nations, economic and population expansion in addition to urbanization have escalated the need for water. Regrettably, the availability and supply of water for this increased population have remained constant or even declined as a result of climate change. SDG 6 recognizes that sustainable water management is far more than simply providing clean water and sanitation. It also addresses the broader context of water, which includes water quality and wastewater management, water scarcity and water use performance, water resource management, and the protection and recovery of water-related ecology.^[14]^

### 3.2. Improving access to good drinking water

In 2015, 91% of the global populace had sufficient clean water, surpassing the Millennium Development Goal of 88%. However, over 660 million individuals have a deficit of safe drinking water, especially residents of rural areas in SSA.^[12]^ Over $250 billion in gross domestic product is lost yearly in developing countries as a result of inadequate water and sanitation facilities. At the national level, this figure can reach 7% of GDP. Even for those who have access to water, the service is most times inadequate or unsustainable, and water from improved sources can also nonetheless be unhealthy to drink.

### 3.3. Enhancing access to good sanitation facilities

Only 68% of the global populace has access to enhanced sanitation, far below the Millennium Development Goal of 77%. Sustainable Development Goal 6 targets to ensure enhanced sanitation for all and end open defecation, which pollutes water bodies and spreads illnesses such as cholera, diarrhea, and dysentery. Most deaths from diarrhea are caused by polluted water sources, poor sanitation, and poor hygiene. This problem is more prevalent in rural areas in developing countries. Seven out of ten individuals who lack access to safe and sanitary facilities are residents in rural settings, majorly SSA and South Asia rural settings.^[12]^ In addition, fast urbanization contributes to the fact that more than 700 million individuals resident in the city lack access to sanitation.

Recently, Merid and colleagues^[19]^ carried out research to evaluate the effect of enhanced water access and good hygiene in curbing diarrhea in infants under 5 years old in rural in low- and middle-income nations. This study found that enhanced hygiene was linked with a 16% decline in diarrhea incidence in children under 5 years old. This report is consistent with research conducted in East African countries,^[20]^ Ethiopia,^[21]^ and Tanzania.^[22]^ A systematic meta-evaluation performed by Wolf and colleagues^[23]^ reported that enhanced hygiene interventions could lower the frequency of pediatric diarrhea in developing nations by 24%. A related study in Ethiopia found that children living in homes without toilets had a 1.50 to 4.8 higher risk of having diarrhea than children living in homes with toilets.^[21,24]^ Additionally, a study in a rural setting in Tanzania found that better waste management lowered the likelihood of diarrhea in children by 63%.^[22]^ Enhancing access to clean water, good sanitation, and proper hygiene is a vital arbitration towards enhancing infants’ health care by thwarting the emergence of infectious illnesses. Reduced access to clean WASH systems are the leading source of fecal-oral spread diseases, especially diarrhea, which remains a global public health concern. Apart from diarrhea, reduced WASH additionally aids a heightened threat of some other diseases like malaria, polio, and neglected tropical diseases.^[25,26]^ Enhancing WASH aids the realization of many SDGs. It contributes to improvement in child health (SDG 3), decreasing malnourishment (SDG 2), eradicating juvenile poverty (SDG 1), building conducive working environments (SDG 8), and protecting the environment and climate alteration (SDG 13).^[26]^ While safeguarding global access to clean water, enhanced sanitation, and proper hygiene for everyone is an example of the UN SDGs, its coverage remains low in developing countries.^[12]^

In addition, access to quality water also reduces the incidence of pediatric diarrhea. According to Wolf et al^[23]^ improved drinking water supply reduces the risk of diarrhea by 52%. In a related study, Ko SH et al^[27]^ reported that children under 5 years old living in households drinking treated water in rural Myanmar were less likely to develop diarrhea. Treated drinking water has a negligible chance of containing pathogens that cause diarrhea.

Additionally, access to enhanced sanitation and clean water services has a remarkable influence in reducing diarrhea. According to Merid et al^[19]^ 24.5% of diarrhea incidence in infants beneath 5 years of age could be reduced through access to enhanced sanitation and quality water in developing countries. Improve water and sanitation services and improve diarrhea prevention. Fuller et al^[28]^ corroborated this in their study which aimed to investigate the combined impact of clean water and good sanitation on diarrhea incidence. This study shows that both interventions have a greater impact on reducing diarrhea than improving water and sanitation alone.^[28]^

## 4. Discussion of findings

This narrative review adds to an already existing broader knowledge of the impact of WASH interventions in lowering diarrhea morbidity and mortality in children aged 5 and below in low-and-middle-income countries. This review serves to consolidate the available publications and therefore provides valuable insights about the effect of WASH interventions on the child health outcomes and what these imply for the achievement of SDG 3 and other cross-linked SDGs.

This review pinpointed a strong scientific body of knowledge proving that WASH interventions can cause a decrease in the number of morbidity and mortality cases due to diarrhea for children younger than 5 years in underdeveloped countries. Multiple studies indicated in the review that diarrhea incidents were greatly reduced owing to the access to clean water, sanitation facilities, and hygiene. For instance, Merid et al^[19]^ observed that increased water availability and good hygiene contributed to a reduction in incidence of diarrhea by 16 percent among children under 5 years old in rural, low- to middle-income countries. Consequently, according to Wolf et al,^[23]^ hygiene strategies, for example, be able to cut down the frequency of childhood diarrhea by almost a quarter in poor countries. These observations concur with the past researches that accentuated the impact of WASH approaches for preventing diarrheal diseases. These diseases remain a main cause of morbidity and mortality among the children in resource-limited areas. This review involves critically synthesizing and analyzing the available evidence that in turn, strengthens the existing literature on the effectiveness of WASH interventions, and at the same time supports the integration of WASH strategies in broader public health program to improve child health outcomes. On the other hand, the results of this overview emphasize the significance of WASH actions in fulfilling the targets of SDG 3 and other SDGs. The provision of clean water, sanitation facilities and hygiene practices not only improves child health but also contributes to the broad range of development goals such as the fight against malnutrition (SDG 2), poverty eradication (SDG 1), community building (SDG 11) and environmental preservation (SDG 13). It is also necessary to consider the factors limiting the included studies, including differences in research designs, methodologies, and settings, which influence the applicability of the evidence to different groups. Similarly, the findings of this review aid in pinpointing the effectiveness of WASH interventions through revealing appropriate implementation procedures, making programs scalable and sustainable in various socio-economic contexts.

## 5. Conclusion

In conclusion, this narrative review has, therefore, provided useful knowledge regarding WASH interventions as they relate to the reduction of diarrhea morbidity and mortality in children under aged 5 years in LMICs. The main finding of the review showed that WASH interventions have big bearing on the health of children where several studies observed a reduction in the incidence of diarrhea following the availability of clean water, sanitation, and hygiene practices.

### 5.1. Implications

Similarly, these results are significant for public health policy and implementation, whereby WASH measures represent a current front line of defense in the campaign against diarrheal diseases, whose overall burden is the chief cause of child illness and death in resource-poor settings. Moreover, encompassing WASH within appointed programs is cardinal for accomplishment of SDG 3 (Sustainable development Goal 3) and other linked SDGs such as those focusing on eradication of poverty, reduction of malnutrition, sustainable community undertakings and protecting the environment.

### 5.2. Directions for future research

This review provides a helpful foundation, however, there are paths for future research endeavors to add to our understanding on the efficiency and implementation of WASH interventions. On the one hand, research with a more advanced methodology should be aimed at finding out the long-term impact and feasibility of WASH programs, taking into consideration different social and economic conditions. Moreover, studies centered on the best application, replication, and cost-effectiveness of WASH interventions in policy and practices are also needed. Also, research examining how the additive effects of bringing WASH interventions together with other public health interventions like nutrition programs and infectious disease control efforts can be done. The interdisciplinary approach allows nearly all the interventionist effects to be fully realized and the root causes of child health and well-being can be lessened.

On the last, the sustainability of living the investments in WASH projects is indispensable in improving child health and reaching the global development goals. Policy makers and practitioners can lay a solid foundation towards conducting these goal through increased accessibility to clean water, sanitation facilities and hygiene methods. A remarkable improvement will then be witnessed runners up being the vulnerable populations.

## Author contributions

**Conceptualization:** Esther Ugo Alum.

**Methodology:** Esther Ugo Alum, Emmanuel Ifeanyi Obeagu, Okechukwu Paul-Chima Ugwu.

**Resources:** Esther Ugo Alum.

**Software:** Emmanuel Ifeanyi Obeagu.

**Supervision:** Emmanuel Ifeanyi Obeagu.

**Validation:** Emmanuel Ifeanyi Obeagu.

**Visualization:** Esther Ugo Alum, Emmanuel Ifeanyi Obeagu.

**Writing – original draft:** Esther Ugo Alum, Emmanuel Ifeanyi Obeagu, Okechukwu Paul-Chima Ugwu.

**Writing – review & editing:** Esther Ugo Alum, Emmanuel Ifeanyi Obeagu, Okechukwu Paul-Chima Ugwu.
